# Pulmonary adverse drug event data in hypertension with implications on COVID-19 morbidity

**DOI:** 10.1038/s41598-021-92734-7

**Published:** 2021-06-25

**Authors:** Majid Jaberi-Douraki, Emma Meyer, Jim Riviere, Nuwan Indika Millagaha Gedara, Jessica Kawakami, Gerald J. Wyckoff, Xuan Xu

**Affiliations:** 1 1DATA Consortium, Manhattan, USA; 2 Kansas State University Olathe, Olathe, KS 66061-1304 USA; 3 Department of Mathematics, Kansas State University, Manhattan, USA; 4 Division of Pharmacology and Pharmaceutical Sciences, School of Pharmacy, University of Missouri-Kansas City, Kansas City, USA; 5 Kansas State University, Manhattan, USA; 6 North Carolina State University, Raleigh, USA; 7 Department of Business Economics, University of Colombo, Colombo, Sri Lanka; 8 Molecular Biology and Biochemistry, School of Biological and Chemical Sciences, University of Missouri-Kansas City, Kansas City, USA

**Keywords:** Infectious diseases, Hypertension, Health policy, Public health, Applied mathematics, Computational science, Computer science, Statistics

## Abstract

Hypertension is a recognized comorbidity for COVID-19. The association of antihypertensive medications with outcomes in patients with hypertension is not fully described. However, angiotensin-converting enzyme 2 (ACE2), responsible for host entry of the novel coronavirus (SARS-CoV-2) leading to COVID-19, is postulated to be upregulated in patients taking angiotensin-converting enzyme inhibitors (ACEIs) and angiotensin II receptor blockers (ARBs). Here, we evaluated the occurrence of pulmonary adverse drug events (ADEs) in patients with hypertension receiving ACEIs/ARBs to determine if disparities exist between individual drugs within the respective classes using data from the FDA Spontaneous Reporting Systems. For this purpose, we proposed the proportional reporting ratio to provide a statistical summary for the commonality of an ADE for a specific drug as compared to the entire database for drugs in the same or other classes. In addition, a statistical procedure, multiple logistic regression analysis, was employed to correct hidden confounders when causative covariates are underreported or untrusted to correct analyses of drug-ADE combinations. To date, analyses have been focused on drug classes rather than individual drugs which may have different ADE profiles depending on the underlying diseases present. A retrospective analysis of thirteen pulmonary ADEs showed significant differences associated with quinapril and trandolapril, compared to other ACEIs and ARBs. Specifically, quinapril and trandolapril were found to have a statistically significantly higher incidence of pulmonary ADEs compared with other ACEIs as well as ARBs (*P* < 0.0001) for group comparison (i.e., ACEIs vs. ARBs vs. quinapril vs. trandolapril) and (*P* ≤ 0.0007) for pairwise comparison (i.e., ACEIs vs. quinapril, ACEIs vs. trandolapril, ARBs vs. quinapril, or ARBs vs. trandolapril). This study suggests that specific members of the ACEI antihypertensive class (quinapril and trandolapril) have a significantly higher cluster of pulmonary ADEs.

## Introduction

The renin-angiotensin system (RAS) is a complex pathway that regulates, among other things, blood pressure and cardiovascular remodeling^1^. Nearly half of adults in the United States have hypertension and angiotensin-converting enzyme inhibitors (ACEIs) and angiotensin II receptor blockers (ARBs) are recommended as first-line agents in non-Black patients with hypertension, making use of these medications widespread^2–4^. Angiotensin-converting enzyme 2 (ACE2) is a counter-regulatory carboxypeptidase of the RAS and the cellular receptor responsible for the viral entry of SARS-CoV-2^5^. ACE2 is predominately expressed in the heart, intestine, kidney, and pulmonary alveolar cells and it has been postulated that ACE2 is upregulated in patients taking ACEIs/ARBs^5–7^.

Following the outbreak of a novel beta coronavirus, later coined SARS-CoV-2, in Wuhan, China in late 2019, numerous questions have emerged regarding the effect comorbidities and their associated medications—including ACEIs and ARBs—may have on the virulence and clinical course of the infection. Patients with underlying comorbidities, including hypertension, were more likely to die from COVID-19, although this correlation is complicated by auxiliary comorbidities and advanced age^5,7,8^. Another study, despite noting that patients with COVID-19 were more likely to have cardiovascular disease, did not find that the use of ACEIs or ARBs independently increased the risk of contracting SARS-CoV-2^7^. ACEIs or ARBs administered before hospital admission were not associated with worse clinical outcomes for patients with COVID-19^9^. Importantly, studies to date probing drug effects on COVID-19 pathogenesis only report drug-class effects and not on individual drugs within a class. In fact, our recent investigation of RAS inhibitors in a dataset of patients with diabetes identified only captopril as having a unique cluster of multiple pulmonary adverse drug events (ADEs) that could impact the pulmonary symptomology of COVID-19^10^. We hypothesized that disparities exist between individual drugs within the respective classes in patients with hypertension receiving ACEIs/ARBs. Therefore, the present study reports on a retrospective analysis of curated ADE databases to evaluate the occurrence of a cluster of pulmonary ADEs in patients with hypertension taking specific ACEIs or ARBs^11^.

## Results

A total of 296,359 ADEs were reported by the FDA’s Adverse Event Reporting System (FAERS) from the first quarter of 2004 to the last quarter of 2019. These data were filtered to isolate pulmonary ADEs totaling 8687 for ACEIs (captopril, lisinopril, quinapril, ramipril, enalapril, perindopril, fosinopril, cilazapril, benazepril, trandolapril) and 1440 for ARBs (azilsartan, irbesartan, losartan, olmesartan, telmisartan, valsartan). We further delineated these data to include thirteen pulmonary ADEs (pulmonary edema, pleural effusion, oropharyngeal pain, dyspnea, dysphonia, cough, sinusitis, pneumonia, nasopharyngitis, bronchitis, pneumonia aspiration, emphysema, and pleurisy) based on association with acute pulmonary illnesses, which resulted in ADE reports totaling 3292 for ACEIs and 1290 for ARBs (total = 4582)^12–17^.

**Figure 1:**
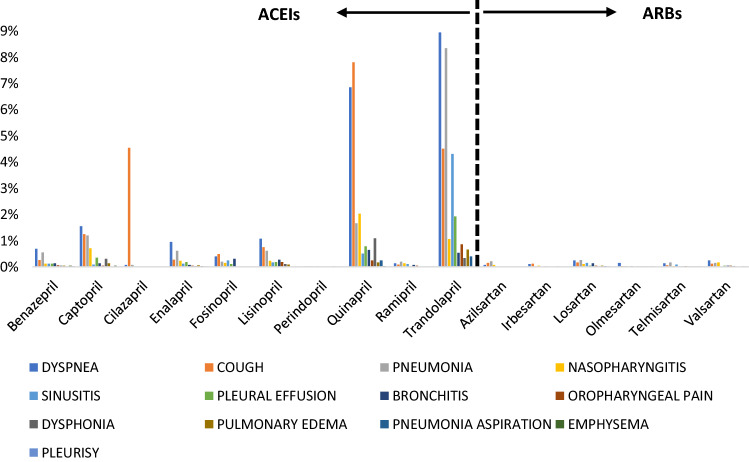
Percentage of the total number for a specific pulmonary ADE divided by total ADEs (n = 4582) from the first quarter of 2004 to the last quarter of 2019 reported for ACEIs and ARBs in patients with hypertension.

Figure 1 illustrates the percentage of the total number for a specific pulmonary ADE divided by total ADEs reported for all ACEIs and ARBs studied (see also Figs. S1 and S2 in SUPPLEMENTARY DATA). As seen in Table 1, the Friedman test indicated that the pulmonary ADEs associated with quinapril, an ACEI, was statistically significantly different compared to ACEIs-beta ($p < 0.001$; ACEIs-beta is all ACEIs except quinapril and trandolapril) as well as ARBs ($p = ~0.0007$). Trandolapril, another ACEI, was statistically significantly different compared to ACEIs-beta ($p = ~0.0001$). Because quinapril and trandolapril were found to be statistically significant from the ACEI class, they were removed from the ACEI class to compare against others in the class and ‘ACEIs-beta’ is used to represent all ACEIs except quinapril and trandolapril. The results indicated that all the seven comparative analyses were significant, especially when comparing ACEIs-beta versus ARBs versus quinapril versus trandolapril ($p~ < ~0.0001$), except for the ACEIs-beta versus ARBs ($p~ = ~0.1481$) and quinapril versus trandolapril ($p~ = ~0.1864$).

**Table 1:** The results of Friedman tests comparing seven pairwise/groups were calculated using ACEIs-beta, ARBs, quinapril, and trandolapril.

Pairwise/group comparison	*P*-value
ACEIs-beta versus ARBs versus quinapril versus trandolapril	< 0.0001
ACEIs-beta versus ARBs	0.1481
ACEIs versus ARBs	0.81
ACEIs-beta versusquinapril	< 0.0001
ACEIs-beta versus trandolapril	0.0001
ARBs versus quinapril	0.0007
ARBs versus trandolapril	0.0004
Quinapril versus trandolapril	0.1864

*ARBs* angiotensin II receptor blockers, *ACEIs-beta* angiotensin-converting enzyme inhibitors excluding quinapril and trandolapril.

**Figure 2:**
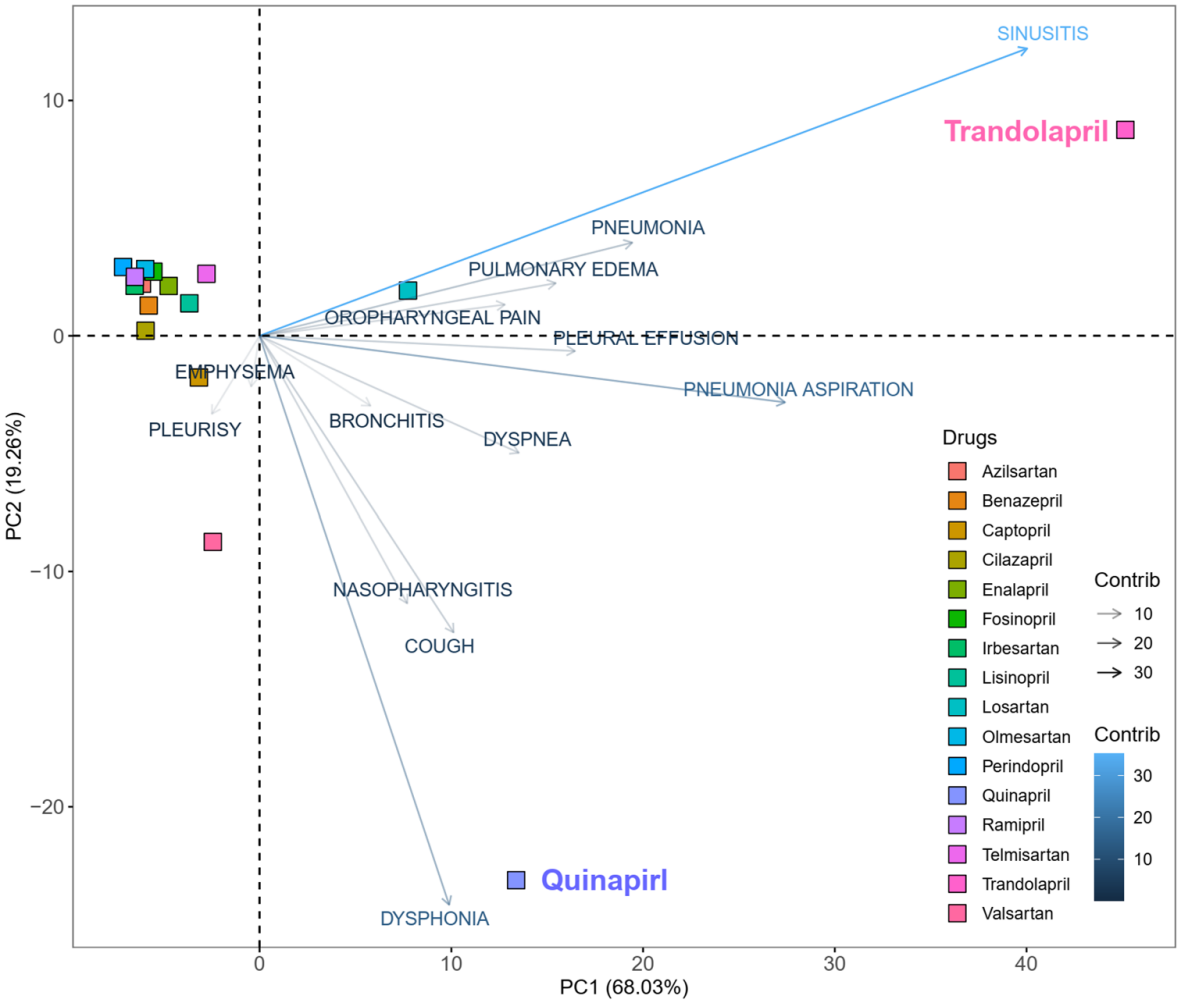
Principal component analysis of proportional report ratios for ACEIs and ARBs.

Figure 2 depicts the optimal representation of two active variables (ADEs) in biplots acquired by PCA and correspondence analysis, which diminishes the effect of supplementary variables that have no or little influence on the ACEI/ARB drugs. The first and second principal components, PC1 and PC2, explaining approximately 90% of variation are presented by the two axes of variation in the proportional reporting ratio (PRR) of ACEIs and ARBs and account for 68.03 and 19.26% of the variation, respectively. Arrows are used to reflect all the variables of pulmonary ADEs, and filled circles show drugs using different colors. The cluster pattern of ACEI and ARB drugs shows three groups: quinapril, trandolapril, and the other ACEIs-beta and ARBs as one group. This results in a triangle shape where each group occupies a different vertex of the triangle. Also depicted in Fig. 2, sinusitis and pneumonia aspiration have the largest positive loadings on PC1 (pointing to the positive direction of PC1), while dysphonia, cough, and nasopharyngitis have the largest negative loadings on PC2 (pointing downward in the negative direction of PC2).

**Figure 3:**
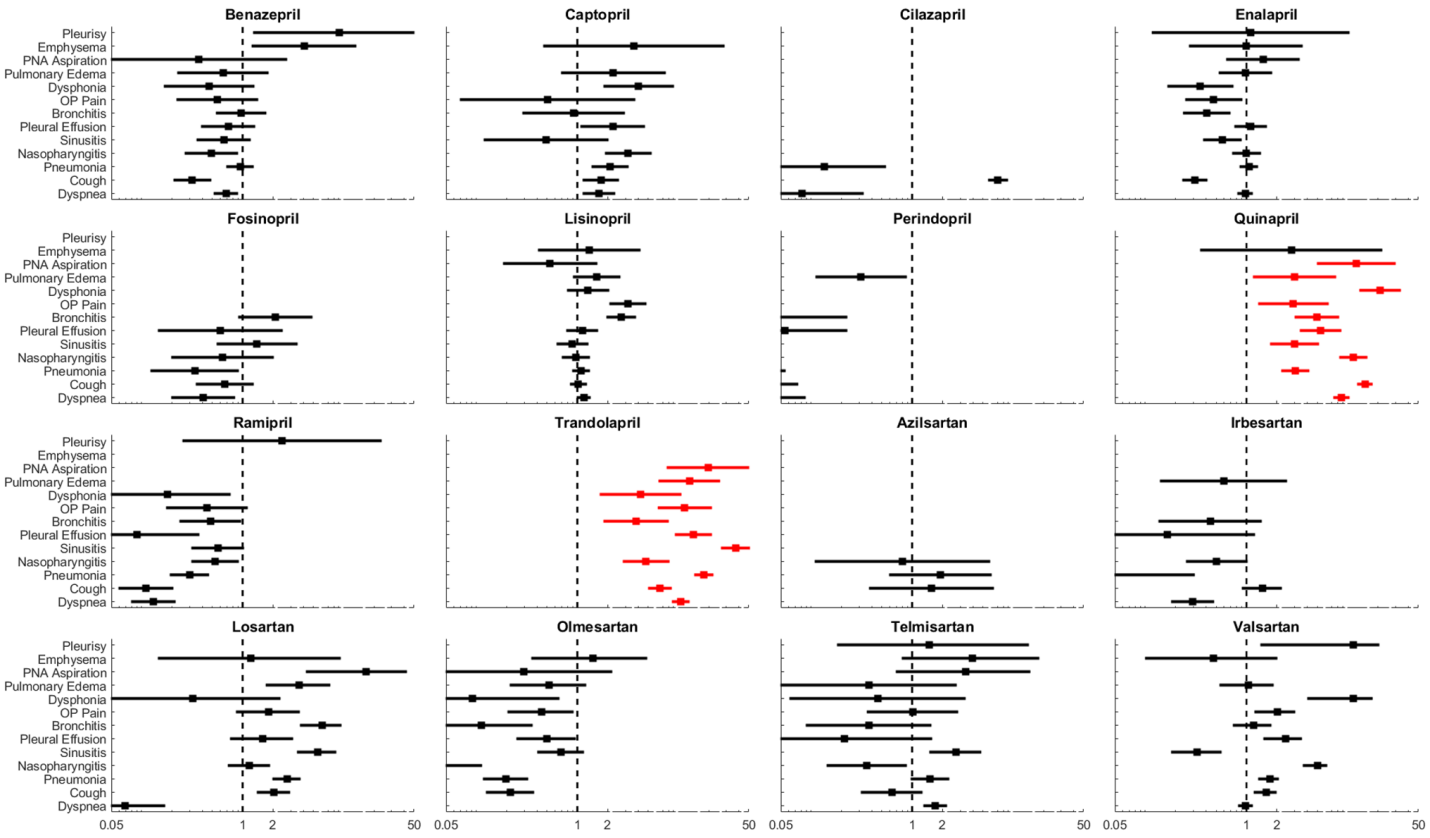
PRR ranges and corresponding confidence intervals for the pulmonary ADEs associated with specific ACEI and ARB drugs. Quinapril and trandolapril associated pulmonary ADEs (shown in red) are significantly different from the other fourteen drugs. The dashed line in each panel corresponds to PRR $\user2{~} = 1$. The abbreviations in the y-axis labels for PNA and OP correspond to pneumonia and oropharyngeal, respectively.

Table 2 shows the results of PRR for each pulmonary ADE associated with each drug against the same ADE from other drugs in the same or different class. The results of each PRR for sixteen ACEI/ARB drugs are obtained from Eqs. (1) and (2) as compared to the ratio factor 1, shown by the black dashed lines representing the mean values of PRRs in each panel of Fig. 3, and more than 3 occurrences reported for each drug-ADE combination in Table 2.

**Table 2:** ADEs (flagged bold) meeting criteria for reporting.

Drug	Outcome												
	Dyspnea	Cough	Pneumonia	NP*	Sinusitis	PE*	Bronchitis	OP*	Dysphonia	PO*	PA*	Emphysema	Pleurisy
Benazepril	1	1	1	1	1	1	1	1	1	1	0	**3**	2
Captopril	2	2	**3**	**3**	0	**3**	1	0	**3**	1	0	1	0
Cilazapril	0	**3**	0	0	0	0	0	0	0	0	0	0	0
Enalapril	1	1	1	1	1	1	1	1	1	1	1	1	0
Fosinopril	1	1	1	1	1	0	2	0	0	0	0	0	0
Lisinopril	2	1	1	1	1	1	**3**	**3**	1	1	1	1	0
Perindopril	1	1	0	0	0	0	0	0	0	1	0	0	0
Quinapril	**3**	**3**	**3**	**3**	**3**	**3**	**3**	**3**	**3**	**3**	**3**	1	0
Ramipril	1	1	1	1	1	0	1	1	0	0	0	0	1
Trandolapril	**3**	**3**	**3**	**3**	**3**	**3**	**3**	**3**	**3**	**3**	**3**	0	0
Azilsartan	**3**	0	1	0	0	0	0	0	0	0	0	0	0
Irbesartan	1	1	0	1	0	0	1	0	0	0	0	0	0
Losartan	1	**3**	**3**	1	**3**	1	**3**	1	0	**3**	**3**	0	0
Olmesartan	0	1	1	0	1	1	1	1	0	1	0	1	0
Telmisartan	2	1	1	1	**3**	0	0	1	0	0	1	1	0
Valsartan	1	2	2	**3**	1	**3**	1	**3**	**3**	1	0	0	**3**

Numbers in the table indicate how many criteria of these three are met: criteria (1) more than 3 occurrences, criteria (2) a PRR > 2, and criteria (3) a PRR that is > than the lower 95% confidence interval boundary with the lower confidence interval being greater than one^18^. ADEs meeting all three criteria are flagged bold.

*NP nasopharyngitis, *PE* pleural effusion, *OP* oropharyngeal pain, *PO* pulmonary edema, *PA* pneumonia aspiration.

## Discussion

This retrospective analysis resulted in three points to consider—first when conducting multifactor analyses across clinical databases containing complex disease processes, individual drugs rather than drug classes should be assessed as ADE profiles can vary in a statistically significant manner. Second, ADEs are generally studied based on individual symptoms (i.e., dyspnea), which may mask patterns of symptoms reflecting dysfunction of a specific organ system (i.e., respiratory). Two ACEIs in this study, quinapril and trandolapril, were found to have a statistically significant difference in reported pulmonary ADEs that should be taken into account during a pandemic displaying pulmonary symptomology. Third, our results prompt consideration of the etiology responsible for the differences in pulmonary ADEs of quinapril and trandolapril in comparison to other ACEIs. A previous study completed by the authors found that in evaluating these drugs in a dataset of patients with diabetes, only captopril had a statistically significant difference in pulmonary ADEs^10^—suggesting that underlying disease etiology may play a role in ADEs. Patients with hypertension commonly have comorbid conditions, which makes correlating specific patterns of ADEs difficult. Ultimately, it is important to realize that individual drugs—not entire classes—can potentially worsen concurrent pulmonary diseases, such as COVID-19, complicated even further by the complex, time-dependent, and divergent symptomology of COVID-19 itself.

One limitation of the present study is that it is a retrospective analysis of curated ADE databases from spontaneous reporting systems and nuances in reporting could affect our datasets. Because this project uses data voluntarily reported to the FAERS and MedDRA databases, it is unknown if the patterns depicted in our data are due to true underlying etiologies or simply, reporting patterns. Prevalence of hypertension is another major limitation, as evidenced by the fact that 29% of all Americans over the age of 18 have hypertension, but that number dramatically increases to 63.1% for American adults over the age of 60^19^. This natural confounding of age and hypertension is a frequent limitation to discerning the impact RAS medications may have on the COVID-19 clinical course, made more difficult by the fact that older adults are more likely to be affected by both hypertension as well as COVID-19^7^. And, the most common causes of pulmonary disease are environmental effects- this is a confounding factor that cannot be addressed using ADE data.

Covariates such as population sampling stratification (for covariates of age, weight, and sex,) are known to affect studies as it has been observed that the ADEs we examine are present as symptoms across all age, weight, and sex groups of patients with COVID-19^20,21^. This effect may attribute to the behavior of the whole system of drug-ADE combinations and the causal relationships within strata data or individual subpopulation, which may lead to creating selection bias^22^. Recently, there has been an increased interest in introducing different methods based on the propensity matching score to reduce or eliminate such effects when using observational data^23^. However, after implementing logistic regression for the known covariates, we were able to correct the analysis and combine this approach with PRR to improve the analysis of drug effects in the hypertension data sets. In our assessment, we observed that there are no apparent negative effects caused by the confounding factors of age, weight, and sex groups, they seem to be extraneous variables that do not affect the PRR analysis of individual drugs versus drug classes. We found that ADEs such as dysphonia, bronchitis, and pleurisy are not significantly affected by any of these covariates.

Several other confounding variables in the design and conduct of studies concerning spontaneous reporting systems can introduce destructive bias or variation. With limited information, a meta-analysis of the observational evidence about the source, magnitude, and effect of these factors remains inconclusive^22,24–26^. Origin of variation and bias in spontaneous reporting systems studies can be identified from clinical features, demographic characteristics, disease morbidity, prevalence and severity, effect and presence of verification bias, arbitrary time-dependent causing bias reports, sampling variance effects, endogenous selection bias conditioning on a collider variable, and time-varying confounding in observational research^22,27–30^. Clinical features and outcomes of patients and demographic characteristics as discussed previously may lead to variations in estimates of test performance, possible age‐ or sex-associated bias creating flaws in clinical reasoning as the other element of sampling error from reporting bias due to systematic nonobservation^22,31,32^. There have been various attempts to eliminated coverage bias using statistical adjustments including weighting or modeling methods to minimize or eliminate multiple sources of bias due to nonobservation error. This demonstrates several biases might have interfered with making a correct determination in our study. Another source of bias might come from sampling variance that addresses the variance of the sampling distribution for reporting rates that commonly fluctuate across drugs, ADE reports, and time and is associated with many factors. It estimates the spread or variability of the ADE-drug combination about its expected value in hypothetical repetitions. Sampling variance constitutes one of the main components of sampling error associated with our ADE database that does not cover the entire population.

Other bias sources include selection biases occurring from the non-random selection of patients exposed to the drug or inaccurate selection of contributors from distorted spontaneous reports. These sources may be driven by covariates other than the drug of interest in the study (e.g., a patient’s pulmonary disease stage or disease duration). These sub-optimal selections may refer to the disproportionality investigation to link a drug and ADEs when confounding equivalence in a covariate is missing causing a synthetic association. A signal attributable to synthetic associations occurs when a drug is inexplicably associated with an ADE that is more properly linked to the underlying disease. For example, it is common for hypertension drugs to be reported with vision loss or blood in the urine which are not usually the effects of treatment. We have addressed some of these bias-associated issues we faced by eliminating the effect of demographic, drug, and ADE stratification that helped mitigate the effect of covariates, without the requirement to distribute reports into multiple strata, and thus extends the applicability and power of existing methods.

Very few studies have analyzed the comparative potencies of ACEIs, and none have categorized quinapril and trandolapril together—and distinct from other ACEIs—as seen in our analysis of ADEs. It should be noted that it is their metabolites, quinaprilat or trandolaprilat (respectively), that are the active moieties in vivo. Hayase et al. 2003 reported that quinaprilat and trandolaprilat had the highest lipophilicity compared to other ACEIs, which could be responsible for increased lung penetration and therefore, increased pulmonary ADEs. This study also investigated their protection from damage affected by lysophosphatidylcholine (LPC) and found that these two ACEIs significantly reduced the LPC-induced hemolysis compared to other drugs in this class^33^. However, this study did not look at ADEs related to these drugs and we have not examined the link between the ADE observed and the similarities in lipophilicity^33^.

Our results emphasize that there are disparities of reported pulmonary ADEs between drugs within the same class, even though most drugs are typically clinically grouped by their class. It is possible that conflicting data regarding the effect ACEIs/ARBs may have on SARS-CoV-2 infection is, in part, due to drugs being evaluated by class instead of individually, and that studies do not take into account different underlying comorbidities. In a recent study by Sablerolles et al.^9^, treatment with ACEIs/ARBs prior to admission for COVID-19 treatment, there was no association with the use of these classes of drugs with clinical outcome. Importantly, this study coded drugs only by class and not individual drugs eliminating any potential to tease out the adverse pulmonary events of the specific drugs we have documented in this study^9^. Despite statistically significant differences of pulmonary ADEs reported for trandolapril and quinapril compared to other ACEIs as well as in comparison to ARBs, more research is needed to determine the clinical significance of this finding. Although this study found important distinctions between drugs both within and between classes, it does not provide a direct clinical recommendation that any of these medications—especially those recommended as first-line antihypertensive agents by the 2017 ACC/AHA guidelines^4^—be discontinued when a patient is diagnosed with COVID-19. The role of ACEI and ARB medications in COVID-19 mortality and morbidity is a complex and still debated topic, especially when considering the benefit these medications have on cardiovascular and renal function. This study does not intend to promote termination of these maintenance medications but instead suggests that further research is warranted to determine if the risk of pulmonary ADEs differs within the ACEI and ARB drug classes and if switching to a different drug within each class is associated with decreased risk of pulmonary symptoms.

## Methods

### Definition of adverse events

The Food and Drug Administration (FDA) defines the term ‘adverse event’ as: “any untoward medical occurrence associated with the use of a drug in humans, whether or not considered drug related, including the following: an adverse event occurring in the course of the use of a drug product in professional practice; an adverse event occurring from drug overdose whether accidental or intentional; an adverse event occurring from drug abuse; an adverse event occurring from drug withdrawal; and any failure of expected pharmacological action”^34,35^.

### Multidimensional database sources

The data used in this study have been curated from multiple publicly available data sources for patients with hypertension, including the FDA’s Adverse Event Reporting System (FAERS), which houses all ADEs reported to the FDA by pharmaceutical companies, healthcare providers, and consumers. The data, including the hypertension dataset, is updated quarterly by the FDA and currently includes reports submitted from the first quarter of 2004 to the last quarter of 2019. This dataset focuses on drugs and their ADEs but includes additional data such as disease, drug, and demographic information as well as information related to patient outcome.

The data structure of these ADEs is organized in accordance with the Medical Dictionary for Regulatory Activities (MedDRA) terminology, along with the International Safety Reporting Guidance Database. We utilized the MedDRA hierarchy for regulatory information of medical products in hypertension, which is grouped based on etiology, manifestation site, or purpose. Here we utilized the 23.0 or earlier version of MedDRA, with the most recent update from April 2020 that includes new COVID-19 related terms and revisions.

### Data mining and search strategy

In alignment with our previous multidisciplinary work^10,36^, we implemented a three-stage approach to curate disparate databases and identified patients with hypertension excluding pulmonary arterial and intracranial hypertension. First, data mining and annotation algorithms using regular expression were used to identify hypertension datasets and associated post-marketing ADEs for ACEI/ARB drugs that were prevalent among the top reported symptoms in patients with COVID-19^37,38^. Since various sources and countries generate phrases and terminologies for each data field in the FAERS database, the drug names (e.g., generic names, brand names, international names, medicinal products, active substances, or active ingredients) contained several mismatched phrases, (international) special characters, and typoes. We used Natural Language Processing (NLP) techniques for extracting information and minimizing the exclusion of similar drugs or ADEs^39,40^. This was done by implementing partial matching algorithms, Levenshtein distance methods, and regular expressions applied to cluster drugs with the same active substance after parsing^41–43^. Levenshtein distance methods were used for various reported drug names in variant spellings to automatically create the most comprehensive drug list. Next, as part of data cleaning, standard libraries were utilized to curate missing information or unify distinct groups within the data, including using manual curation following MedDRA terminology as well as the International Council for Harmonization of Technical Requirements for Pharmaceuticals for Human Use^10^. For example, drug names in the FAERS database are reported by a combination of active ingredients, generic names, or brand names. Using PostgreSQL (PostgreSQL Global Development Group) allowed us to map and search all the possible drug names to drug parents in the DrugBank database (Alberta Innovates—Health Solutions, The Metabolomics Innovation Centre) creating a unified dataset^44^. Additionally, ADEs derived from unstructured data (e.g. text) needed data scrubbing, cleansing, and merging^18^. For this purpose, deep learning techniques were employed to implement and map the informatic structure of the FAERS database into the international safety reporting guidance coded using terms in MedDRA^18^. The semantic similarity of drugs was processed by using regular expressions when cleaning and merging drug information from different sources. Drug information in FAERS records includes brand names, generic names, active substances, and active ingredients, drugs with the same active substances were merged while irrelevant drugs were eliminated from the analysis. Finally, ADEs associated with medications in the ACEI and ARB classes administered to patients with hypertension were recorded.

### Proportional reporting ratio

Statistical analysis was performed using SAS (SAS University Edition version 9.4, North Carolina, U.S). First, data based on the frequency of each ADE related to respiratory, thoracic, and mediastinal disorders/infections were parsed in the MedDRA and FAERS databases. Specific ADEs collected were pulmonary edema, pleural effusion, oropharyngeal pain, dyspnea, dysphonia, cough, sinusitis, pneumonia, nasopharyngitis, bronchitis, pneumonia aspiration, emphysema, and pleurisy (Fig. 1). These ADEs were consistent with globally reported information, which found that pneumonia, pneumonitis, shortness of breath, cough, and sore throat were among the top reported symptoms in patients with COVID-19^12–17^. Emphysema was included in our study as it was suggested by several studies and CDC guidelines for people with certain medical conditions^45–47^. Also, a recent study reported that pulmonary emphysema may mimic COVID-19 in combination with other conditions^48^. We then employed a method proposed and implemented by the FDA for analyzing ADE disproportionality in pharmacovigilance data by observed-expected ratios^18^. This method, the proportional reporting ratio (PRR), provides a statistical summary for the commonality of an ADE for a specific drug as compared to the entire database for drugs in the same or other classes^18^.

Patient demographics and drugs that are under-reported in voluntary reporting systems, including the FAERS, were addressed since conditional slicing and sub-setting can confine the use of quantitative signal detection methods such as PRR. It must be noted that not all potential confounding factors can be addressed: for example, some drugs are used for multiple conditions, and not all co-morbidities can be addressed based on the available ADE data using this analysis. However, we were able to correct the analysis after applying logistic regression for the known covariates of age, weight, and sex, and combine this approach with PRR to improve analyses of drug effects using the hypertension data sets. As a result, we found that the following identity is chiefly correct in numerous scenarios:

$$\Pr \left( {{\text{ADE|drug}},{\text{age}},{\text{weight}},{\text{sex}}} \right) = {\text{~Pr}}(A{\text{DE}}|{\text{drug}})$$

This helped us to estimate a PRR for a specific drug-ADE combination by calculating the following equation:

1 $${\text{PRR}}_{{ij}} = \frac{{{\text{Pr}}(ADE_{i} |drug_{j} )}}{{{\text{Pr}}(ADE_{i} |drug_{j}^{*} ~)}} = \frac{{\frac{{r_{{ij}} }}{{n_{j} }}}}{{\frac{{\left( {\mathop \sum \nolimits_{{k = 1}}^{L} r_{{ik}} ~~~ - r_{{ij}} } \right)}}{{\mathop \sum \nolimits_{{i = 1}}^{D} \mathop \sum \nolimits_{{k = 1}}^{E} r_{{ik}} ~~ - n_{j} }}}}$$

where $r_{{ij}} ~$ gives the total number of a specific ADE $i \in \left\{ {1,2, \ldots ,E} \right\}$ for a given drug $j$ in $\left\{ {1,2, \ldots ,D} \right\}$. Here $E$ and $D$ represent the number of all events and drugs in the drug class, respectively. $drug_{j}^{*}$ denotes the drug class, excluding the specific drug $j$. Also, $n_{j}$ shows the total events for the given drug $j$. As the distributions of PRR samples are all positive, we then applied a log transformation to data and found the confidence interval^49^ using the following equation:

2 $$95\% ~{\text{CI}}_{{ij}} = \exp \left( {{\text{ln}}\left( {{\text{PRR}}_{{ij}} } \right) \pm 1.96 \times {\text{SD}}_{{ij}} } \right)$$

where

$${\text{SD}}_{{ij}} = \sqrt {\frac{{n_{j} - r_{{ij}} }}{{n_{j} \times r_{{ij}} }} + \frac{{\mathop \sum \nolimits_{{i = 1}}^{D} \mathop \sum \nolimits_{{k = 1}}^{E} r_{{ik}} ~~ - n_{j} }}{{n_{j} \times \mathop \sum \nolimits_{{i = 1}}^{D} \mathop \sum \nolimits_{{k = 1}}^{E} r_{{ik}} ~}}} {\text{~}}$$

### Friedman test results

Using SAS, sample differences among the four groups—quinapril, trandolapril, ACEIs, and ARBs—were assessed for a pairwise analysis with the assumption that data were not normally distributed using the non-parametric Friedman test for two independent unequal-sized data. Friedman test was also applied to perform multiple comparison tests (*P* values for statistical significance < 0.05). For the non-parametric Friedman test of statistical significance, seven pairwise and multiple comparisons were performed based on the ARBs and ACEIs excluding quinapril and trandolapril hence denoted as ACEIs-beta. Tests performed included ACEIs-beta versus ARBs, ACEIs-beta versus quinapril alone, ACEIs-beta versus trandolapril alone, quinapril versus ARBs, trandolapril versus ARBs, and quinapril and trandolapril versus all ACEIs-beta and ARBs.

### Principle component analysis

Principal components of PRR for pulmonary ADEs associated with ACEIs and ARBs were calculated using the built-in function *prcomp* in R 3.6 (R Core Team, GNU GPL v2)^50^. Implementing principal component analysis (PCA) to the drugs with 13 pulmonary ADEs reduced the dimension to a smaller number of PCs, significantly explaining and visualizing variation of ACEIs and ARBs. The biplot, Fig. 2, was generated using the R package *factoextra*^51^.

### PRR ranges and corresponding confidence intervals

The result of PRR ranges and corresponding confidence intervals for the thirteen ADEs and sixteen ACEI and ARB drugs shown in Fig. 3. was implemented in MATLAB R2019b (MathWorks Inc., Natick, MA, USA). Line plots and error-bars were depicted with MATLAB’s built-in *errorbar* function. The scale on the *x*-axis was transformed to logarithm form for improved interpretation of the PRR ranges using *Axes* properties function.

## Supplementary Information

Supplementary Information.

## Data Availability

All the data supporting the findings in this study are available here (https://figshare.com/articles/Data_for_HTN_Pulmenoary_ADE_Reports/12555116) and in Supplementary Information. Data related to this paper are available from the corresponding authors upon request.
