# Supplementary material for: Pulmonary adverse drug event data in hypertension with implications on COVID-19 morbidity
Source: Sci Rep. 2021 Jun 25;11:13349. doi: 10.1038/s41598-021-92734-7 (PMC8233397; doi:10.1038/s41598-021-92734-7)
Supplement: Supplementary file 1 — Supplementary Information. [file 41598_2021_92734_MOESM1_ESM.docx]

**TITLE:** Pulmonary Adverse Drug Event Data in Hypertension with Implications on COVID-19 Morbidity

Majid Jaberi-Douraki^1,2,3,^ *****, Emma Meyer^1,4^, Jim Riviere^1,5^, Nuwan Indika Millagaha Gedara^1,2,6^, Jessica Kawakami^1,4,7^, Gerald J. Wyckoff^1,4,7^, Xuan Xu^1,2^

^1^1DATA Consortium, [www.1DATA.life](http://www.1DATA.life), USA

^2^Kansas State University Olathe, Olathe, KS 66061-1304

^3^Department of Mathematics, Kansas State University

^4^School of Pharmacy, Division of Pharmacology and Pharmaceutical Sciences, University of Missouri-Kansas City
^5^Kansas State University and North Carolina State University
^6^Department of Business Economics, University of Colombo, Sri Lanka

^7^Molecular Biology and Biochemistry, School of Biological and Chemical Sciences, University of Missouri-Kansas City

**^*^Correspondence to email address:** [jaberi@k-state.edu](mailto:jaberi@k-state.edu)

**SUPPLEMENTARY DATA**

**Fig. S1.** Percentage of the total number for a specific pulmonary ADE reported for ACEIs in patients with hypertension.

**Fig. S2.** Percentage of the total number for a specific pulmonary ADE reported for ARBs in patients with hypertension.
